# Word reading skills in autism spectrum disorder: A systematic review

**DOI:** 10.3389/fpsyg.2022.930275

**Published:** 2022-07-27

**Authors:** Ana Paula Vale, Carina Fernandes, Susana Cardoso

**Affiliations:** ^1^Dyslexia Unit, Department of Education and Psychology, School of Social and Human Sciences, University of Trás-os-Montes e Alto Douro, Vila Real, Portugal; ^2^Laboratory of Neuropsychophysiology, Faculty of Psychology and Education Sciences, University of Porto, Porto, Portugal

**Keywords:** autism, word reading strategies, decoding, word recognition, methodological features

## Abstract

A growing body of research suggests that children with autism spectrum disorder (ASD) are at risk of reading and learning difficulties. However, there is mixed evidence on their weaknesses in different reading components, and little is known about how reading skills characterize in ASD. Thereby, the current study aimed to systematically review the research investigating this function in children with ASD. To this purpose, we reviewed 24 studies that compared (1) children with ASD and children with typical development (TD) in word and nonword reading performance, (2) children with ASD and normative data of word and nonword reading tests, and (3) the results obtained by children with ASD in word and nonword reading tests. Most of the comparisons (62%) contrasting the reading performance of children with ASD and children with TD did not find significant differences between groups in both word and nonword reading. However, all the comparisons that reported standardized results showed that children with ASD had scores that fell within population norms. Regarding the third comparison of interest, about 54% of the studies presented data for both word and nonword reading, but only one study tested the difference between them and showed that children with ASD had higher levels of word than of nonword reading. Despite these results, the heterogeneous and small samples do not allow to draw sound conclusions regarding the strategies that children with ASD use to read words. As consequence, the nature of reading difficulties presented by children with ASD are still unknown, requiring future research conducted with larger and well-characterized samples of ASD and TD, using homogeneous specific tasks designed to assess word reading strategies.

## Introduction

A majority of students with autism spectrum disorder (ASD) has reading difficulties (Ricketts et al., 2013; McIntyre et al., 2017b; Solari et al., 2019). Indeed, there is evidence that even when children and adolescents with ASD perform at the normative range of general cognitive ability and are verbally able, only a small percentage (31.2%) has average reading scores. This data contrasts with what is generally shown in typically developing (TD) peers of similar general cognitive ability, in which at least 80% achieve average scores (Solari et al., 2019). This indicates that a large number of ASD students are not responding well to reading instruction and/or reading instruction may not be well-designed to enable their reading success. One way or another, this argues for the need to understand how children with ASD deal with reading.

Autism spectrum disorder is a neurodevelopmental disorder with early-onset described by some degree of impairment in social interaction and communication. Other characteristics include restricted and repetitive behaviors and interests. Crucially, these features have a detrimental impact on daily life (American Psychiatric Association, 2013; World Health Organization, 2019). Recent ASD prevalence estimates of nationwide and across countries accounts range from 0.8 to 1.5% of school-age children (Lyall et al., 2017; ASDEU, 2018; Baio et al., 2018; Ofner et al., 2018) reported by the Centers for Disease Control and Prevention surveillance network for autism in the USA, being that two-thirds of 8-years old children with ASD did not present intellectual disability (Maenner et al., 2021). Thus, it is conceivable that most children with ASD may be enrolled in core curriculum educational programs to learn how to read (Fleury et al., 2014). Reading has a critical role in adapting to current and future academic, cognitive, and social needs and challenges (Lyon, 2001; Maughan et al., 2020).

Many studies have examined reading skills in ASD (Davidson and Weismer, 2014; Dynia et al., 2014; Bednarz et al., 2017; Nally et al., 2018; Micai et al., 2021). Among studies of reading skills and development in ASD, the majority paid particular attention to children's difficulties in reading comprehension, that is, their struggles to obtain meaning from written passages or texts (Nation et al., 2006; Brown et al., 2013; Ricketts et al., 2013; McIntyre et al., 2017a; Solari et al., 2019). It is often assumed that reading comprehension difficulties of children with ASD derive from oral language limitations (e.g., Nation and Norbury, 2005; Huemer and Mann, 2010; Ricketts, 2011; El Zein et al., 2014; Singh et al., 2021). This has guiding many to assume that readers with ASD have a hyperlexic profile (Fernandes et al., 2015; Ostrolenk et al., 2017; Duncan et al., 2021; Macdonald et al., 2021). That is, they usually present low levels of reading comprehension along with good abilities of word reading. However, it may not be the case (Henderson et al., 2014; Solari et al., 2019; Macdonald et al., 2021). To fully understand the obstacles that children with ASD face to achieve efficient comprehension of written information there is a need to better examine word reading processes in ASD, as much as reading comprehension can be conceived as essentially the same as language comprehension in written format (Hoover and Tunmer, 2020).

According to the Simple View of Reading (SVR, Gough and Tunmer, 1986; Hoover and Tunmer, 2020), a framework of the cognitive capacities needed for reading with wide empirical support (Fernandes et al., 2017a,b; Lonigan et al., 2018; Nation, 2019; Verhoeven et al., 2019; Kim et al., 2021), reading comprehension is the product of two sets of skills: word reading and oral language skills. This means that neither of these two components is sufficient *per se* for achieving reading comprehension and if one of them is somehow failing, reading comprehension will fail too. Thus, SVR establishes that reading difficulties may be dependent on word reading problems, language comprehension problems, or both (Hoover and Gough, 1990).

A recent meta-analysis (Duncan et al., 2021) aiming to clarify the role of these two skills in reading comprehension in ASD computed data of 26 studies that included both a measure of word reading and reading comprehension. Their analyses showed that each of the SVR components made a similar size contribution to the statistical model, demonstrating that word reading is as critical as the oral language to achieve reading comprehension for children with ASD. Other studies had previously shown that word reading has an important role in reading comprehension in ASD (Brown et al., 2013; Ricketts et al., 2013). For example, Brown et al.'s (2013) meta-analysis reported that, although children with ASD had word reading standard scores within the average range, which were better than their weak oral language scores, word reading explained a comparable amount of unique variance as the oral language (57%) in reading comprehension. Moreover, word reading was strongly associated with reading comprehension (*r* = 0.77, *n* = 1,469).

These findings concur with emerging evidence indicating that there is more than one type of reading profile among children and adolescents with ASD. For instance, Solari et al.'s (2019) found four different reading profiles: (a) a group with average word reading and reading comprehension (18%); (b) a group with specific difficulties in reading comprehension (poor comprehenders; 24%); (c) a group with low scores in both word reading and reading comprehension but good receptive vocabulary (23.6%) and (d) a group with a profile of generalized low scores in word reading, reading comprehension and oral language (34.3%). Interestingly, a former study (Henderson et al., 2014) similarly found that only 24.5% of their sample of children and adolescents with ASD could be characterized as poor reading comprehenders, presenting word reading accuracy on the average range, reading comprehension below a standard score of 89 and a discrepancy of at least 1 standard deviation (SD) between the two. In this study, 57% scored more than 1 SD, and 31% more than 2 SD, below the mean on word reading. These and other studies (Nation et al., 2006; White et al., 2006; Jonhels and Sandberg, 2012; McIntyre et al., 2017a) point out that, contrary to the widespread oral language deficit only explanation for reading comprehension problems (Singh et al., 2021), many children and adolescents with ASD have word reading difficulties.

Since the majority of studies on reading in ASD have been designed to address children's reading comprehension difficulties, many enrolled children with average to high levels of word reading (Engel, 2018; Ibrahim, 2020; Macdonald et al., 2022) and did not examine specific effects on single-word reading, such as frequency, orthographic consistency, and length of words. This trend contributed to a scarce knowledge about how children with ASD process written words.

One of the key hypotheses regarding word reading is that its development evolves through the emergence of two broad mechanisms described by the main theories of skilled reading. Essentially, the three most acknowledge computational models of fluent reading—Dual route cascaded model (DRC; Coltheart et al., 2001), the Triangle model (Plaut et al., 1996; Harm and Seidenberg, 2004), and the Connectionist Dual Process model (CDP++; Perry et al., 2010)—agree on a need for two-pathways to read words, independent of the particular orthography to be learnt: a direct, lexical process that merge the words spellings to the meanings usually preferred for familiar words and irregular words (such as *have, come*, and *eye*, that cannot be correctly read using only grapheme - phoneme conversions); and a sub-lexical indirect way *via* the phonological serial conversion of each grapheme into a phoneme, to obtain a pronunciation and then the word meaning, used mostly for new and low frequency words.

Thus, in the same vein, single-word reading is considered to involve at least two main cognitive mechanisms: decoding and recognition (Castles et al., 2018; Miles and Ehri, 2019). Decoding is employed when children use a phonological/alphabetic approach in which a pattern of grapheme—phoneme correspondences are assembled sequentially to sound out a word. This mechanism requires conscious cognitive effort and, consequently, it is also a time-consuming procedure. Recognition, on the other hand, is an almost effortless automatic process of accurately matching a written word with an orthographic pattern stored in long-term memory combined with phonological and semantic information (Miles and Ehri, 2019), called orthographic strategy. As Miles and Ehri (2019) detailed, this strategy does not equate with visual memory processes. Instead, it depends on a tuned representation of the specific string of amalgamated grapho-phonemic structures composing a word that draws on high levels of orthographic knowledge.

At the beginning of reading acquisition TD children rely predominantly on the alphabetic/phonological strategy to decode most of the words they encounter. Gradually, with further instruction and great amounts of exposure they gain sophisticated knowledge about the specificities of the orthographic system functioning and eventually achieve the automaticity that characterizes written word recognition (Castles et al., 2018; Miles and Ehri, 2019). Importantly, decoding is deemed to be a crucial skill to develop written word recognition (Share, 1995). Thus, the strategies children use to read single words adjust to their reading ability (Ehri, 2013).

Data on word reading strategies of children with ASD is very scarce and offers mixed evidence on the relative strengths and weaknesses of word reading skills among these readers.

Some small-scale studies (Frith and Snowling, 1983; Minshew et al., 1995) found that children with ASD who had word reading levels within the expected range for their age were also able of decoding nonwords (a string of letters, such as *slint*, that do not exist in the lexicon and thus is virtually independent of the memory for individual words, requiring decoding skills in order to be read). Later studies have also found good levels of nonword reading in groups of school-age children with ASD (Gabig, 2010; McIntyre et al., 2017b). On the contrary, Nation et al. (2006) noted that many ASD children had difficulties when reading nonwords and White et al. (2006) reported that more than half of their sample of children with ASD presented word decoding difficulties and poor phonological awareness, a skill that enables isolating each phoneme in a word and that is robustly related to word decoding (Melby-Lervåg et al., 2012). More recently, Henderson et al. (2014) found that although word and nonword reading were strongly correlated in a group of children with ASD, nonword reading scores were significantly lower for the ASD group than for a group of their TD peers matched by word reading level. Also Westerveld et al. (2018) found significant floor effects for nonword reading but not for words in first graders with ASD. Thus, because nonwords reading is thought to require the use of the sub-lexical indirect route according to the computational theories of reading mentioned above these results suggests that for many ASD children the indirect/phonological path for reading may present some degree of dysfunctionality and decoding appears to be a challenging task, being unclear how they read unfamiliar words.

Considering the above-mentioned results, we may argue that children with ASD that achieve typical scores on word reading may not be using phonological strategies but instead a direct access procedure based on their visual memory, possibly supported by intact or enhanced associative learning mechanisms (Walenski et al., 2008) and/or enhanced processing of broad visual aspects of written material (Samson et al., 2012; Ostrolenk et al., 2017) along with a detail-focused style of cognitive processing (Happé and Frith, 2006) that may favors word patterns recognition. In line with this, Macdonald et al. (2021) observed preschool children with ASD and hyperlexia exhibiting advanced word reading and letter naming in tandem with low phonological awareness and letter-sound correspondence skills. Other studies (Hooper et al., 2006; White et al., 2006; Gabig, 2010; Jonhels and Sandberg, 2012) have also reported that children with ASD showed poorer phonemic awareness than their age-matched peers, conflicting with Frith and Snowling (1983) findings.

Together, these results suggest that children with ASD may be employing their own strategies to process word reading. However, Cardoso-Martins et al. (2015) reported that a group of Brazilian Portuguese speaking children with ASD, varying considerably in nonverbal intelligence and word reading ability, did not differ from their TD colleagues, matched for word reading accuracy, on nonword reading. In addition, the ASD group presented an equivalent reading accuracy in word and nonword reading. Also, likewise to their TD peers, word reading was strongly correlated with nonword reading for the ASD group. The authors argued that participants with ASD used a similar phonological-based sub-lexical strategy as their peers for reading, which contrasts with the formerly mentioned evidence. In face of this results and in consonance with the Psycholinguistic Grain Size Theory of Reading (Ziegler and Goswami, 2005) mentioned by the authors, we could reason that, since the match between letters and sounds is more fixed in the Portuguese orthography than in the English one, the learning and use of the sub-lexical indirect-phonological route could be easier in Portuguese than in English (Duncan et al., 2013) that often requires reliance on orthographic structures larger than single letters to achieve word reading. Although this was never tested with ASD children, it may not be the unique explanation for the Brazilian results. As a matter of fact, Frith and Snowling (1983) reported a pattern of results similar to those of Cardoso-Martins et al. (2015), showing that ASD English children did not differ from their TD peers on using the sub-lexical procedure better than the lexical one. Thus, it seems that there is a number of discordant findings regarding word reading skills of ASD children challenging the reaching of a coherent description.

A robust predictor of reading automaticity and therefore word recognition (Landerl et al., 2019) is Rapid Naming (RAN). RAN, a task requiring the serial naming of arrays of familiar pictures of objects or colors or letters in a speedy manner, is supposed to involve, like reading, the lexical retrieval of familiar phonological sequences. While there is consistent evidence that children with ASD perform more poorly than their peers presenting longer naming times (White et al., 2006; Gabig, 2010; McIntyre et al., 2017b; Nayar et al., 2021), RAN was shown to be significantly associated with word reading fluency, but not accuracy (Johnels et al., 2021). This suggests that many children with ASD could experience difficulty in building word reading automaticity; that is, difficulty in using the direct/lexical procedure hypothesized by the computational theories of reading (Plaut et al., 1996; Coltheart et al., 2001; Perry et al., 2010).

Although scarcely, other psycholinguistic effects were examined in ASD reading studies. For instance, Welsh et al. (1987) showed a significant frequency effect indicating that ASD children had grew a lexicon for written words and used it successfully. Still, the same children read regular words better than irregular ones suggesting that they were applying grapheme-phoneme conversion rules more effectively than using lexical orthographic knowledge. Earlier work (Frith and Snowling, 1983) had reported the same pattern of results, showing that children with ASD presented an advantage of regular words both in accuracy and time scores. According with the computational theories of reading mentioned above, these frequency and regularity effects point out that some ASD children can use both direct/lexical and indirect/phonological sub-lexical routes to read words. Yet, because the studies have a small number of participants and have large age ranges, this body of findings does not clearly elucidate about the word reading skills of ASD children.

Thus, studies on reading acquisition and development in ASD offer divergent evidence of what might be the word reading strategies of those children. However, knowing how children with ASD read words and, complementarily, identifying the challenges they meet in that endeavor is vital to assist in teaching them to read and in designing solid remediation interventions when they are needed. Furthermore, as word reading is necessary for reading comprehension that, in turn, is determinant to progress in other academic subjects and to increase the knowledge of the world (Hoover and Tunmer, 2020), it is critical to systematize what is the current knowledge concerning the word reading processes used by children with ASD.

## Methods

### Systematic search strategy

This review was performed according to the actualized Preferred Reporting Items for Systematic Reviews and Meta-Analyses (PRISMA) guidelines (Page et al., 2021). Articles published until December 2021 were selected from PubMed, Web of Science, and EBSCOhost (including the Academic Search Complete, Psychology and Behavioral Sciences Collection, CINAHL Plus with Full Text, Fonte Acadêmica, MedicLatina, PsycARTICLES, PsycBOOKS, and PsycINFO databases). The search expression was **“**(autis^*^ OR ASD) AND (read^*^ OR literacy OR “word decoding” OR “word recognition”). In addition, we screened the reference list of reviews in this field and of all included studies.

### Selection criteria

We included experimental and quasi-experimental studies that have assessed word reading abilities in children with autism spectrum disorders (ASD). In this study, we included children aged up to 12 years. Considering that at the age of 6 reading skills are becoming reasonably well-established (Nation et al., 2006; Henderson et al., 2014), an upper limit of 12 years was chosen to avoid ceiling effects.

After being included for reporting research in the topic of the review, articles were excluded according to the following criteria: (a) articles without a group of children with ASD (children with ASD mixed with other diagnosis, children with typical development or participants with diagnosis of ASD with mean age > 12 years old); (criterion 1: wrong population); (b) articles <10 ASD participants (criterion 2: case series); (c) articles that did not assess word reading abilities (criterion 3: wrong measure); (d) inaccessible articles or studies without information about word reading abilities in children with ASD (criterion 4: lack of data); (e) articles published in other languages than English, Portuguese, Spanish, or French (criterion 5: inaccessible language); (f) other orthographic system rather than alphabetic (criterion 6: non-alphabetic system) and (g) abstracts, reviews, commentaries, or methods (criterion 7: wrong publication type). Articles reporting only duplicated data were also excluded, and when articles reported an expansion of previously conducted research, data from the most recent article were selected (criterion 8: duplicated data).

### Screening and selection of records

The results of the literature search were compiled on Rayyan QCRI (Ouzzani et al., 2016). On this platform, two researchers blindly screened the titles and abstracts, excluded the articles out of topic, and retained the remaining studies. When this task was completed, the screening was unblinded. The reference lists of the included empirical studies and reviews were also screened, retaining titles in the topic that did not appear in the systematic search. Two authors read all the retained studies and, independently, decided to include or exclude them. Disagreements in both stages were solved by consensus.

### Data collection and analysis

The data of each included article were added to an extraction sheet developed for this review and refined when necessary. When available, the following variables were extracted from each article: year of publication; diagnostic/inclusion criteria used by the authors to compose the ASD samples; sample size of each group (children with ASD and typically developed controls, when present) and number of female participants; mean age and standard deviation per group; mean years of education and standard deviation per group; name and description of word reading tools or tasks; results obtained to each dependent variable (means and standard deviations per group); *p*-values and direction of the significant differences between groups or conditions.

Considering the goals of this systematic review, the results will be reported by comparison of interest: (1) comparison between children with ASD and children with typical development, and (2) comparison between children with ASD and normative data. Since one of our goals is also to understand the reading strategies of children with ASD, we will also report (3) the comparisons between the results obtained in word and nonword reading.

## Results

**Figure 1:**
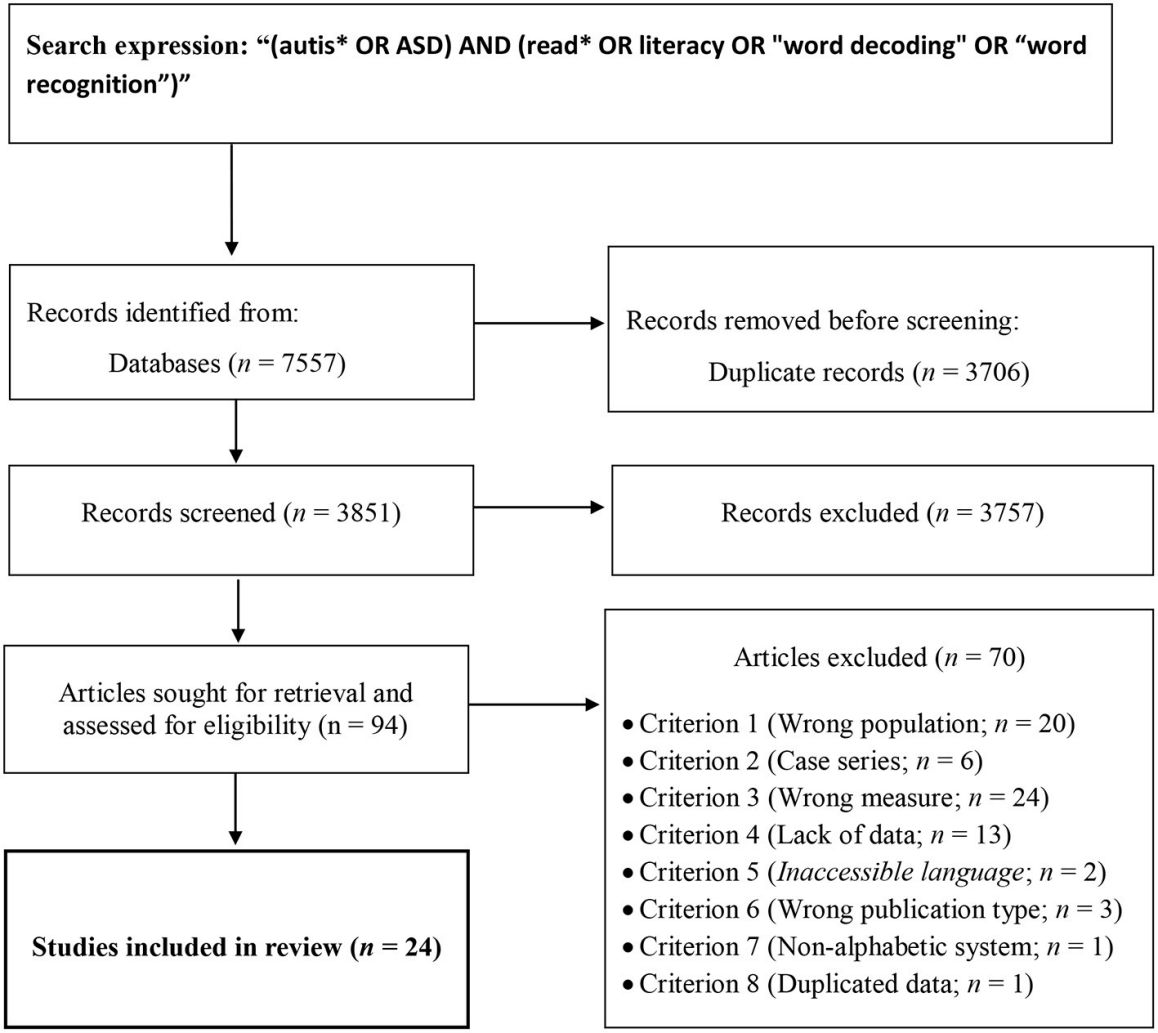
Flow diagram illustrating the systematic search, results and the selection of the studies included in this systematic review.

The systematic search provided 3,444 titles. The search in the reference lists and other sources provided four additional studies. After excluding duplicates, 7,557 studies were screened based on titles and abstracts. A total of 94 articles were selected for full-text assessment of eligibility, and the remaining articles were excluded for being off-topic. From the full text assessment, 24 articles were included in the review. The entire selection process is represented in the flowchart of Figure 1.

The 24 included studies were published between 2006 and 2021 and provide data from 1,549 children with ASD (about 19% females; *M*_*pooledage*_= 7.58*, SD*_*pooledage*_ = 1.10) and from 1,187 children with typical development (about 55% females; *M*_*pooledage*_ = 9.96*, SD*_*pooledage*_ = 1.16). The entire sample of children with ASD was composed of verbal children.

Fifteen studies (62.5%) compared the performance of children with ASD and children with typical development, while nine studies (37.5%) compared the performance of children with ASD with the normative results. In these cases, the raw scores were converted to standardized scores (mean is 100 and standard deviation is 15) or were presented through the percentile. Two study presented longitudinal research, but only baseline data were analyzed (Solari et al., 2019, 2022).

**Figure 2:**
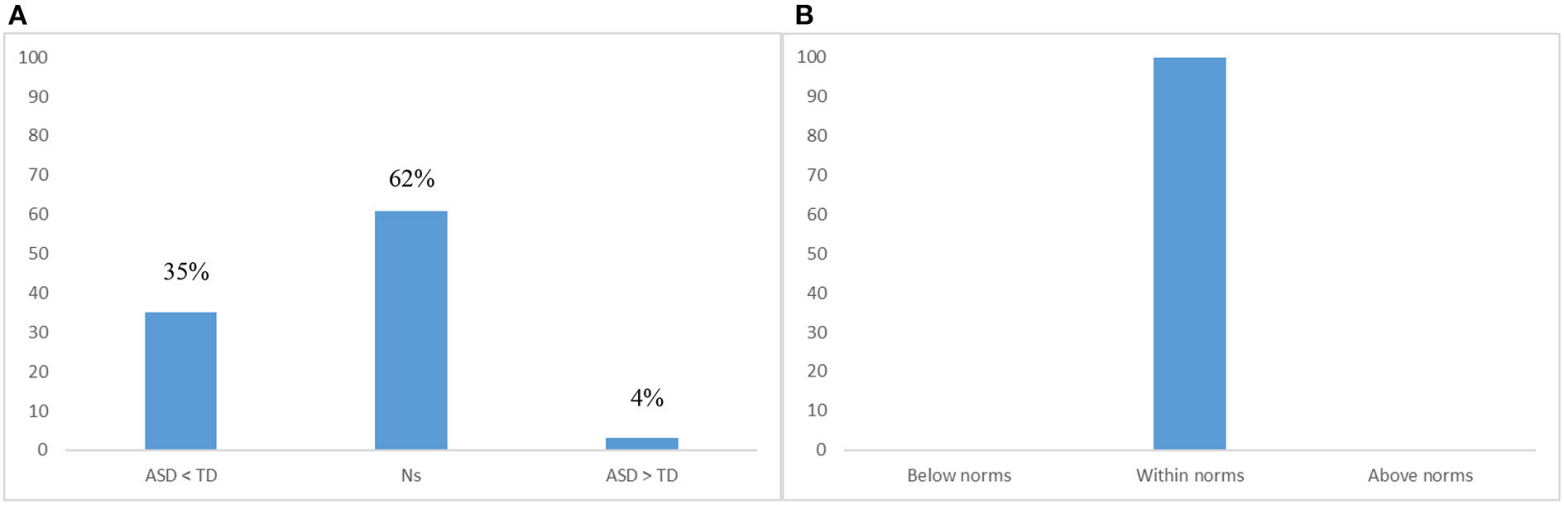
**(A)** Percentage of tests or subtests that compared children with autism spectrum disorders (ASD) and children with typical development (TD); **(B)** Percentage of tests or subtests that compared children with ASD with normative scores or percentiles.

As these studies were composed of several reading tests and tasks, they provided 41 comparisons of interest. Specifically, we found that: 26 tests or subtests were used to compare the performance of children with ASD and children with typical development, while 15 tests or subtests provided standardized data from children with ASD (Figure 2). Of note, we included studies that were designed to assess word reading abilities or word reading strategies in children with ASD (e.g., Nation et al., 2006; Henderson et al., 2014; Cardoso-Martins et al., 2015), and also studies designed with other goals (e.g., Arciuli et al., 2013) but had at least one measure of word or nonword reading in their assessment.

The Test of Word Reading Efficiency (TOWRE; Torgesen et al., 1999) was the most used test, followed by the Woodcock Reading Mastery Tests (WRMT; Woodcock, 2011). Table 1 presents a description of all the tasks identified in the present review, along with their distribution by comparison of interest. The totality of the tests and tasks demanded a verbal response.

**Table 1:** Description of the tests included in this review and the number of comparisons (*N*) in which they were used.

**Test**	**Subtest**	**Test description**	***N*** **ASD vs. TD**	***N*** **standardized scores**
Test of Word Reading Efficiency (TOWRE)	Sight word efficiency (SWE)	Participants read as many real words as they could in 45 s	5	2
	Phonemic decoding efficiency (PDE)	Participants read as many decodable nonwords as they could in 45 s	4	3
H4 test (Franzén, 1997) and LS test (Johansson, 1992)	–	Timed tests of single, out of context, word reading. It assesses word decoding efficiency. The H4 test was used for girls in grades 2–6 (8–12 years), while the LS test was used for the older girls (13–17 years)	1	–
Wordchains test (Jacobson, 1996)	–	Assesses word decoding ability and fluency. The task is to mark with a pencil where divisions should be made in a chain of three words without inter-word blank spaces (e.g., carhousetree). Task duration = 90 s	1	–
Phonological judgment (Auphan et al., 2018)	–	The task is to judge if a word and a pseudoword (a list of pairs) sound equal. Task duration = 2 min	1	–
Woodcock Reading Mastery Tests-Revised (WRMT-R)	Word identification	Assesses the child's ability to recognize sight word vocabulary of increasing difficulty	4	1
	Word Attack	Assesses the ability to phonetically decode pseudowords	4	3
TOWRE and WRMT-R	–	In one study (Lu et al., 2016), word reading was measured with the average of the standard scores of word identification, word attack, sight word efficiency, and phonemic decoding proficiency	1	–
Woodcock Johnson Test of Achievement—IV edition	Letter-Word Identification	Assesses single word reading	1	1
Test of School Performance	Word reading	Comprises 70 words printed in lower-case letters on a card in order of increasing difficulty	1	–
	Nonword reading	Assesses phonological decoding: the child was asked to read 20 pseudowords	1	–
Phonological Awareness Literacy Screening for Kindergarten (PALS-K; Invernizzi et al., 2015)	PALS-K—word Identification	Literacy screening tool that measures kindergarteners' developing literacy skills. The PALS-K—word identification assesses a student's ability to recognize words in text	1	–
Graded Nonword Reading Test (GNWRT; Snowling et al., 1996)	–	Involves reading nonwords presented in isolation	1	1
British Ability Scales (BAS-II; Elliot et al., 1996)	Word reading	Involves reading words presented in isolation that gradually increase in difficulty	1	1
Wide Range Achievement Test-IV	Word reading	Involves reading aloud letters and words	–	1
Illinois test of psycholinguistic abilities (ITPA-3; Hammill et al., 2001)	Sight decoding	Involves reading a list of printed words	–	1

N, frequency of use the respective task; ASD, children with autism spectrum disorder; TD, children with typical development.

Regarding the results of the individual studies, 62% of the comparisons (*n* = 16) contrasting the reading performance of children with ASD and children with TD did not find significant differences between groups in both word and nonword reading. However, 35% of the comparisons (*n* = 9) found that children with ASD had a significantly worse performance than children with TD, while 4% of the comparisons (*n* = 1) found the opposite pattern of results. These results are presented in detail on Table 2. With one exception (Lucas and Norbury, 2014b), the results obtained for word reading were consistent with the results obtained for nonword reading. Only Lucas and Norbury (2014b) found that children with ASD had worse performance than children with TD for word reading, although the groups did not differ significantly in nonword decoding.

**Table 2:** Results of the studies that compared the performance of children with ASD with children with typical development.

**References**	**Participants** ***N*** **(female)**	**Age** ***M*** **(*****SD*****)**	**Reading test**	**Dependent** **variable**	**Effect** **direction**	**Main results**
Johnels and Sandberg (2008)	TD: 19 (3) ASD: 37 (4)	8.81 (1.34) 9.74 (1.87)	Wordchains test	Word reading	Ns	Strong association between word decoding fluency and sentence reading comprehension in ASD group even after the effect of age and Verbal IQ was partialled out.
Johnels et al. (2010)	TD: 54 (54) ASD: 20 (20)	12.5 (2.6) 11.8 (2.7)	H4 test and LS test	Word reading	Ns	The TD and ASD girls performed very close to the normative mean on the literacy tests.
Auphan et al. (2018)	TD: 89 (56) ASD: 10 (1)	10.5 10.27	Phonological judgment	Phonological judgment		Analyses were carried out case by case. 3/10 of children with ASD had word reading decoding difficulties.
Davidson (2016)	TD: 21 (7) ASD: 21 (3)	–	Word Identification—WRMT-III Word Attack—WRMT-III	Word reading Nonword decoding	Ns Ns	^*^
Davidson et al. (2018)	TD: 24 (11) ASD: 19 (4)	10.97 (1.04) 11.21 (1.48)	Word Identification—WRMT-III Word Attack—WRMT-III	Word reading Nonword decoding	ASD < TD ASD < TD	Word decoding was not significantly related to reading comprehension in the TD group. In the ASD group, word decoding significantly correlated with age, reading comprehension, word reading cluster, word recognition and vocabulary.
Gabig (2010)	TD: 10 (3) ASD: 14 (2)	6.8 (0.89) 6.5 (0.72)	Word Identification—WRMT-R-NU Word Attack—WRMT-R-NU	Word reading Nonword decoding	Ns Ns	Children with ASD performed better when decoding words than nonwords: 60% had slow, labored, and inaccurate decoding attempts; 22% attempted to parse the individual graphemes/phoneme relationship and sound out the nonword but could not blend the individual phonemes into a whole; 22% were able to decode the nonwords efficiently.
Henderson et al. (2014)	TD: 49 ASD: 49	–	GNWRT BAS-II	Word reading Nonword decoding	ASD < TD ASD < TD	To examine the discrepancy between word and nonword reading, 25 children with ASD were pair-wise matched to 25 children with TD on raw word reading scores. The ASD group obtained significantly lower nonword reading scores than TD, suggesting that word reading skills are not supported by adequate phonological decoding skills in ASD.
Lu et al. (2016)	TD: 20 ASD: 25	10.3 (3.57) 11.3 (3.48)	TOWRE and WRMT composite score	Word reading	ASD < TD	The reading scores of children with ASD were near the standardized mean of 100, but significantly lower than the scores of the TD group.
Lucas and Norbury (2014a)	TD: 30 (12) ASD-ALN: 25 (3) ASD-ALI: 12 (4)	10.47 (1.01) 11.21 (1.9) 11.77 (1.38)	sight word efficiency—TOWRE phonemic decoding efficiency—TOWRE	Word recognition Nonword decoding	ALI < (ALN = TD) ALI < (ALN = TD)	^*^
Lucas and Norbury (2014b)	TD: 21 (9) ASD: 20 (5)	10.46 (0.92) 10.57 (1.37)	Sight word efficiency—TOWRE Phonemic decoding efficiency—TOWRE	Word recognition Nonword decoding	ASD < TD^a^ Ns	^a^However, groups did not differ significantly when analyzing the raw score of this subtest.
Macdonald et al. (2021)	TD: 15 (11) ASD: 15 (1)	4.08 (0.67) 4.58 (0.83)	Letter-Word Identification	Word reading	ASD < TD	The ASD group was divided in a subgroup of children with and without hyperlexia. This analysis showed that the group with both ASD and hyperlexia exhibited advanced word reading and letter naming skills that TD and ASD without hyperlexia but did not demonstrate commensurate phonological awareness, letter-sound correspondence, or language skills.
Cardoso-Martins et al. (2015)	TD: 19 (0) ASD: 19 (3)	6.5 (0.38) 11.5 (3.9)	Word reading—TDE Nonword reading	Word reading Nonword decoding	Ns Ns	The ability to read and spell words with accuracy was strongly correlated with the ability to read pseudowords in ASD and TD.
McIntyre et al. (2017a)	TD: 44 (16) ASD: 81 (15)	11.59 (2.25) 11.24 (2.19)	Sight word efficiency—TOWRE Phonemic decoding efficiency—TOWRE	Word recognition Nonword decoding	NS Ns	^*^
Solari et al. (2022)	TD: 735 ASD: 616	–	PALS-K—word Identification	Word identification	ASD > TD	^*^
Weissinger (2013)	TD: 37 (18) ASD: 10 (2)	–	Word Identification—WRMT-III Word Attack—WRMT-III sight word efficiency—TOWRE	Word reading Nonword decoding Word recognition	NS NS NS	^*^

ASD, children with autism spectrum disorder; TD, children with typical development; Ns, non-significant differences between groups; WRMT-R-NU, Woodcock Reading Mastery Tests-Revised-Normative Update; TOWRE, Test of Word Reading Efficiency; PALS-K, Phonological Awareness Literacy Screening for Kindergarten; GNWRT, Graded Nonword Reading Test; BAS-II, British Ability Scales; TDE, Teste de Desempenho Escolar [Test of School Performance]; ALN, ASD children with age-appropriate structural language skills; ALI, ASD children with language impairments.

* The article does not provide further qualitative information regarding word reading skills beyond the scores obtained in the reading tests.

Regarding the results of the studies that presented standardized data, the findings were more consistent as 100% of the comparisons showed that children with ASD had scores that fell within population norms. These results are presented in detail on Table 3.

**Table 3:** Results of the studies that compared the performance of children with ASD with normative data.

**References**	**Participants** ***N*** **(female)**	**Age** ***M*** **(*****SD*****)**	**Reading test**	**Language of the study**	**Dependent variable**	**Effect direction**	**Main results**
Arciuli et al. (2013)	21 (3)	7.8 (1.75)	Word Reading—WRAT-IV	English	Word reading	Within population norms	Significant correlation between word-level accuracy and adaptive communication domain of adaptative behavior as assessed by the parent self-report of children's adaptative behavior
Cronin (2014)	13 (2)	9.7	Word Attack—WRMT-III Phonemic decoding efficiency—TOWRE	English	Nonword decoding Nonword decoding	Within population norms Within population norms	No significant correlation between phonology and decoding or comprehension. Strong correlation between semantics and decoding, as well as decoding and comprehension
Jones (2007)	27	–	Word Identification—WRMT-III Word Attack—WRMT-III	English	Word reading Nonword decoding	Within population norms Within population norms	^*^
Johnels et al. (2021)	40	12	Sight decoding—ITPA-3	English	Word decoding	Within population norms	^*^
Knight (2016)	201	–	Word Attack—WRMT-III Letter-Word Identification	English	Word reading Word reading	Within population norms Within population norms	^*^
McIntyre et al. (2017a)	81 (15)	11.24 (2.19)	Sight word efficiency—TOWRE Phonemic decoding efficiency—TOWRE	English	Word recognition Nonword decoding	Within population norms Within population norms	Four profiles of readers: (1) Comprehension Disturbance; (2) Global Disturbance; (3) Severe Global Disturbance; (4) Average Readers. All but the Severe has normative or near normative word reading scores. None manifested a profile of good comprehension and poor word reading
Nation et al. (2006)	32	–	BAS-II GNWRT	English	Word reading Nonword decoding	Within population norms Within population norms	^*^
Quan (2014)	29 (2)	–	Letter-Word Identification	English	Word reading	Within population norms	Majority of children (61%) falling within one SD of population norms; 1 student performed above one SD. Six students (21%) had standard scores below one SD of population norms, three students (11%) fell below two SDs, and one student fell below three SDs
Solari et al. (2019)	80 (15)	11.26 (2.15)	Sight word efficiency—TOWRE Phonemic decoding efficiency—TOWRE	English	Word recognition Nonword decoding	Within population norms Within population norms	Similar reading profiles at time points 1 and 2 of assessment

ASD, children with autism spectrum disorder; TD, children with typical development; Ns, non-significant differences between groups; WRMT-R-NU, Woodcock Reading Mastery Tests-Revised-Normative Update; TOWRE, Test of Word Reading Efficiency; WRAT-IV, Wide Range Achievement Test; ITPA-3, Illinois test of psycholinguistic abilities; GNWRT, Graded Nonword Reading Test; BAS-II, British Ability Scales.

* The article does not provide further qualitative information regarding word reading skills beyond the scores obtained in the reading tests.

About 54% of the studies reviewed (*n* = 13) presented data for both word and nonword reading. Regarding the results, we found that only 4% (*n* = 1) directly tested the difference between them showing that children with ASD had higher levels of word than of nonword reading. Adding to these results, it was noticeable that both word and nonword reading fell within the normal range values in 11 of those studies, being that nonwords presented slightly smaller standardized values than words in 5 studies, slightly bigger in 3 and virtually the same in another 3. In the other two studies, the raw data presented was converted in percentages which allowed to observe that nonwords had lower values than words in one study and nearly the same in the other. These results are presented in detail on Table 4.

**Table 4:** Results of the studies that compared the performance of children with ASD in tests assessing word and nonword reading.

**References**	**Participants** ***N*** **(female)**	**Age** ***M*** **(*****SD*****)**	**Reading test**	**Language** **of the** **study**	**Dependent** **variable**	**Word** **reading**	**Nonword** **decoding**	**Comparison** **word vs.** **nonword**
Davidson (2016)	21 (3)	–	Word Identification—WRMT-III Word Attack—WRMT-III	English	Word reading Nonword decoding	102.90 (12.57)	95.14 (13.91)	Not tested
Davidson et al. (2018)	19 (4)	11.21 (1.48)	Word Identification—WRMT-III Word Attack—WRMT-III	English	Word reading Nonword decoding	98.42 (13.26)	89.47 (11.97)	Not tested
Gabig (2010)	14 (2)	6.5 (0.72)	Word Identification—WRMT-R-NU Word Attack—WRMT-R-NU	English	Word reading Nonword decoding	115 (10.3)	104 (11.2)	ASD: Words > Nonwords TD: NS
Henderson et al. (2014)	49	–	BAS-II GNWRT	English	Word reading Nonword decoding	69.56 (12.58)/90	14.92 (6.92)/25	Not tested
Jones (2007)	27	–	Word Identification—WRMT-III Word Attack—WRMT-III	English	Word reading Nonword decoding	100.17 (15.54)	96.41 (24.08)	Not tested
Lucas and Norbury (2014a)	ALN: 25 (3) ALI: 12 (4)	11.21 (1.9) 11.77 (1.38)	sight word efficiency—TOWRE phonemic decoding efficiency—TOWRE	English	Word recognition Nonword decoding	ALN: 104.69 (12.63) ALI: 91.83 (8.09)	ALN: 109.02 (12.10) ALI: 94.89 (12.03)	Not tested
Lucas and Norbury (2014b)	20 (5)	10.57 (1.37)	sight word efficiency—TOWRE phonemic decoding efficiency—TOWRE	English	Word recognition Nonword decoding	95.48 (13.11)	101.13 (16.94)	Not tested
Cardoso-Martins et al. (2015)	19 (3)	11.5 (3.9)	Word reading—TDE Nonword reading	Portuguese	Word reading Nonword decoding	46.5 (20.43)/70	11.89 (5.71)/20	Not tested; ASD > phonological errors in reading
McIntyre et al. (2017a)	81 (15)	11.24 (2.19)	Sight word efficiency—TOWRE Phonemic decoding efficiency—TOWRE	English	Word recognition Nonword decoding	93.29 (14.75)	94.89 (14.81)	Not tested
Nation et al. (2006)	32	–	BAS-II GNWRT	English	Word reading Nonword decoding	96.56 (23.37)	90.83 (17.87)	Not tested; 64 % of children was 1 SD below norms on nonword reading
Weissinger (2013)	10 (2)	–	Sight word efficiency—TOWRE Word Identification—WRMT-R-NU Word Attack—WRMT-R-NU	English	Word recognition Word reading Nonword decoding	92.00 (11.039) 101.40 (15.63)	111.3 (20.7)	Not tested
Solari et al. (2019)	80 (15)	11.26 (2.15)	Sight word efficiency—TOWRE Phonemic decoding efficiency—TOWRE	English	Word recognition Nonword decoding	94.87 (14.91)	93.66 (14.47)	Not tested

ASD, children with autism spectrum disorder; TD, children with typical development; ALN, ASD children with age-appropriate structural language skills; ALI, ASD children with language impairments; WRMT-III, Woodcock Reading Mastery Tests-Third Edition; WRMT-R-NU, Woodcock Reading Mastery Tests-Revised-Normative Update; TOWRE, Test of Word Reading Efficiency; GNWRT, Graded Nonword Reading Test; BAS-II, British Ability Scales; TDE, Teste de Desempenho Escolar (Test of School Performance).

## Discussion

In the present review we analyzed empirical research in order to systematize the current knowledge concerning the word reading processes used by children with ASD. Twenty-four articles published from 2006 to December 2021 were selected and three data comparisons were analyzed: (1) comparisons between children with ASD and children with typical development regarding word and nonword reading, (2) comparisons between children with ASD and normative data, and (3) comparisons between the results obtained in word and nonword reading in the ASD group.

Considering the comparisons with the typical development peers, it was possible to observe that although a majority of them have shown that the children with ASD achieved similar levels of performance, a considerable percentage (35%) showed lower reading levels. However, 10 out of 15 of those comparisons were conducted with small samples (between 10 and 21 participants), which may have affected the statistical power of the results. The effect of small samples may explain the divergence between the actual results and those found by Solari et al. (2019) study, which indicated that 58% of their 80 participants showed word reading difficulties. In addition, there is evidence that even when there are no significant differences between children with ASD and their typically developing peers on reading accuracy and speed, results from psychophysiological measures (such as eye fixations and regressions) have been showing that reading is often a more effortful task for them than for their colleagues (Howard et al., 2017).

In turn, the totally of the studies that reported standardized measures showed that the reading levels of children with ASD were within the normal range. Although the findings of these two type comparisons are not paradoxical, they should be interpreted with caution. First, it indicates that the presence of a control group seems to be critical to better understand the challenges that children with ASD face when reading. Moreover, putting the methodological precision aside, using only population norms to characterize the average reading profiles may be rather imprecise since the individual variability among children with ASD is large as the standard deviations presented indicate (see, for instance, Table 4).

Analyzing the comparisons between word and nonword reading performances among children with ASD aimed to investigate the reading strategy(ies) more successfully used by these children—decoding (using phonological sub-lexical units) and/or recognition (using lexical patterns). It is important to note that reading words may be achieved by recognition or by the sequential process of converting graphemes into phonemes and assembling them to pronouncing the word—the decoding strategy. It depends on the familiarity of the word and the reading level of the reader. Thus, when authors present data on word reading most of the time there is ambiguity about the cognitive processes in course when children underwent the task. Some tests, however, are more evident. For instance, when it is said that children were asked to read sight words or visual vocabulary it is more likely they were using word recognition than decoding. To the contrary, when children are said to be reading nonwords, they will need to decode them using sub-lexical procedures in order to pronounce them since nonwords does not exist in the mental lexicon and cannot profit from memory for words. Indeed, unless the reader is very unskilled, words require less of decoding than nonwords do (Weekes, 1997) and nonwords cannot be recognized.

As it was mentioned, only 13 studies reported both the results that assessed word and nonword reading. From this small pool of studies, it seems that children with ASD can use both lexical and sub-lexical phonological knowledge to read at a similar easy. It is noteworthy though that there was a patent paucity of direct assessment of the processes underlying word reading performances since all but four studies (Nation et al., 2006; Gabig, 2010; Henderson et al., 2014; Cardoso-Martins et al., 2015) aimed to study other aspects of reading, like comprehension or the relationship between ASD characteristics and comprehension processes. Thus, the characteristics of items to be read were not specifically designed for comparing word with nonword reading and this limits the soundness of the outcome of these comparisons.

Nevertheless, some of the studies suggest that children with ASD may be more prone to rely on recognition processes than in decoding ones. This appears so when, in addition to the reading scores, it is pointed out that children with ASD produced more phonological errors when reading (Cardoso-Martins et al., 2015) and presented a higher percentage of nonword reading levels below the norms, although they did not differentiate from their peers with typical reading levels in word and nonword reading (Nation et al., 2006). Indeed, there is good evidence that children with ASD are skillful in visual patterns processing (Ostrolenk et al., 2017), which can aid their lexical orthographic learning. On the other hand, there is also some research indicating that phonological processes and alphabet knowledge seem to be areas of strength for children with ASD (Frith and Snowling, 1983; Lucas and Norbury, 2014b; Dynia et al., 2016).

These apparent discrepancies suggest that word reading strategies in ASD are far from being well-understood and that studying them should consider environmental variables like reading instruction and the orthographic consistency of the language to be read. Indeed, it is important to point out that the teaching methods the children undertook have probably influenced their performances. Although scarce, there is evidence that children with ASD may have limited instructional conditions (Spector and Cavanaugh, 2015). These authors concluded that children with ASD had a reading instructional time of 60–30 min per day, contrary to recommendations of 90–120 min for K-3 students. Also, there were reports of an instructional overemphasis on narrow skills such as sight-word knowledge which, with time passing, has becoming more combined with code instruction. In the studies reviewed there were no references to teaching methods. Regarding the orthographic consistency, the degree to which each letter has one or more phonological correspondences, and considering that orthographies differ greatly in their consistency and therefore in their correspondent easy to learn to read (Duncan et al., 2013), it is remarkable that we have found only one study that was not run in English, the Brazilian Portuguese study of Cardoso-Martins et al. (2015). Having studies of ASD children's word reading skills run in orthographies more consistent than English, the most inconsistent orthography, is necessary because it is not possible to have a clear idea of reading in ASD without contrasting different orthographies. As it is put by the Psycholinguistic Grain Size Theory of Reading (Ziegler and Goswami, 2005), it is possible that in more consistent orthographies ASD children could, like their TD peers, learn how to read faster and easier than in English showing a different and clearer pattern when considering the lexical and sub-lexical pathways.

Related with the lack of solid data on word reading strategies in ASD is the confusing language of many studies when referring to word reading. In analyzing the selected studies, it was witnessed a generalized interchangeable use of the words “decoding” and “recognition”, as if they were synonyms, to the point that it was often impossible to understand exactly what the authors were talking about. For instance, the phrase “accuracy in word recognition was measured for both real words and for nonwords” is a unique extreme example of incorrectness, but the profusion of inaccuracy and ambiguity in the usage of “decoding” and “word recognition” is probably contributing to curtail a clear understanding on word reading strengths and challenges in ASD.

Given the goals of the research reviewed it is unsurprising that, almost all the studies selected children with ASD and, at least, some reading knowledge. Nevertheless, since there are estimates of about 30% of children with ASD being nonverbal or minimally verbal (Tager-Flusberg and Kasari, 2013), what is somewhat unexpected is that all the studies analyzed required the children to orally respond to the reading tasks. However, some individuals with ASD can read and write meaningfully despite not using spoken language (Goh et al., 2013) and minimally verbal children with ASD were proven to be able to learn to read words and to discriminate between words and nonwords (Serret et al., 2017).

Thus, in addition to little solid evidence about the word reading strategies of children with ASD, this review highlighted the dearth of knowledge about nonverbal children with ASD reading profiles. All the studies included in the review had participants who were verbal and required a verbal response to the reading assessments, and this may be a major limitation of the previous research in this field. Moreover, this fact may explain the evidence that all the reviewed studies that compared the performance of children with ASD with normative data fell within the population norms. If confirmed by future studies, the evidence of a lack of difference between children with ASD and normative data may be a sample effect instead of the true effect of ASD in word reading. Thereby, our results show that the current research is very limiting for understanding autism functioning and diversity. As challenging as it may be, this calls for further research employing valid assessment reading tools that can fairly be used with a larger extent of ASD heterogeneity like silent reading tests and methods like eye-tracking that do not demand verbal answers which may also be conducted with nonverbal children.

Another issue regarding the weaknesses of the results analyzed is that the female samples are very under-represented, considering the known ratio male-female being 3:1 (Loomes et al., 2017). Future studies may take this ratio into account during participants recruitment to constitute samples that are as representative as possible of the real world. This lack of representativeness, associated with the fact that the samples of ASD children were treated as wholes, instead of being grouped according to relevant characteristics (such as oral language level, attentional difficulties, years of schooling, among others), suggests that different results could be shown if these constraints were controlled.

Therefore, future studies should be conducted with a larger number of gender matched participants, using tasks specifically designed to study word reading. They also should include a typical developing control group and collect psychophysiological data, such as eye-tracking measures, to increase the understanding of behavioral outcomes. In addition, samples of children with ASD should be specifically characterized in order to better realize how the natural variability of ASD concur with word reading performances.

This review suggests that children with ASD may have preserved word reading abilities, being a further step in the direction of a better understanding of autism associated word reading challenges, identifying weaknesses of existing studies and opening new directions for future research. However, given the weaknesses found, it is not possible to identify which strategies children with ASD use better to identify written words, nor can we thoroughly deduce what are exactly the word reading difficulties and strengths of children with ASD.

## Data availability statement

The raw data supporting the conclusions of this article will be made available by the authors, without undue reservation.

## Author contributions

AV conducted the literature review, did the systematic search and the inclusion of the articles, analyzed the data, wrote the introduction and the discussion, and revised the manuscript. CF did the systematic search and the inclusion of the articles, analyzed the data, wrote the method, and revised the manuscript. SC analyzed the data, wrote the discussion, and revised the manuscript. All authors read and approved the final manuscript.

## Conflict of interest

The authors declare that the research was conducted in the absence of any commercial or financial relationships that could be construed as a potential conflict of interest.

## Publisher's note

All claims expressed in this article are solely those of the authors and do not necessarily represent those of their affiliated organizations, or those of the publisher, the editors and the reviewers. Any product that may be evaluated in this article, or claim that may be made by its manufacturer, is not guaranteed or endorsed by the publisher.
